# Effects of CO_2_ limitation on the metabolism of *Pseudoclostridium thermosuccinogenes*

**DOI:** 10.1186/s12866-020-01835-2

**Published:** 2020-06-08

**Authors:** Jeroen Girwar Koendjbiharie, Wilbert Berend Post, Martí Munar Palmer, Richard van Kranenburg

**Affiliations:** 1 Corbion, Gorinchem, Netherlands; 2 Laboratory of Microbiology, Wageningen University, Wageningen, Netherlands

**Keywords:** CO_2_ limitation, *Pseudoclostridium thermosuccinogenes*, Fermentation products, Succinic acid, Transcriptomics, Redox balance, Batch fermentation, Chemostat

## Abstract

**Background:**

Bio-based succinic acid holds promise as a sustainable platform chemical. Its production through microbial fermentation concurs with the fixation of CO_2_, through the carboxylation of phosphoenolpyruvate. Here, we studied the effect of the available CO_2_ on the metabolism of *Pseudoclostridium thermosuccinogenes*, the only known succinate producing thermophile. Batch cultivations in bioreactors sparged with 1 and 20% CO_2_ were conducted that allowed us to carefully study the effect of CO_2_ limitation.

**Results:**

Formate yield was greatly reduced at low CO_2_ concentrations, signifying a switch from pyruvate formate lyase (PFL) to pyruvate:ferredoxin oxidoreductase (PFOR) for acetyl-CoA formation. The corresponding increase in endogenous CO_2_ production (by PFOR) enabled succinic acid production to be largely maintained as its yield was reduced by only 26%, thus also maintaining the concomitant NADH re-oxidation, essential for regenerating NAD^+^ for glycolysis. Acetate yield was slightly reduced as well, while that of lactate was slightly increased. CO_2_ limitation also prompted the formation of significant amounts of ethanol, which is only marginally produced during CO_2_ excess. Altogether, the changes in fermentation product yields result in increased ferredoxin and NAD^+^ reduction, and increased NADPH oxidation during CO_2_ limitation, which must be linked to reshuffled (trans) hydrogenation mechanisms of those cofactors, in order to keep them balanced. RNA sequencing, to investigate transcriptional effects of CO_2_ limitation, yielded only ambiguous results regarding the known (trans) hydrogenation mechanisms.

**Conclusions:**

The results hinted at a decreased NAD^+^/NADH ratio, which could ultimately be responsible for the stress observed during CO_2_ limitation. Clear overexpression of an alcohol dehydrogenase (*adhE*) was observed, which may explain the increased ethanol production, while no changes were seen for PFL and PFOR expression that could explain the anticipated switch based on the fermentation results.

## Background

Succinic acid produced by microbial fermentation is an attractive platform chemical, with the potential to contribute to a bio-based economy. A set of different bio-based platform chemicals, such as succinic acid, allows the sustainable synthesis of the majority of our materials [1]. It is important to produce those chemicals as efficiently as possible, especially as our current market requires them to directly compete with cheap and unsustainable fossil fuel-derived alternatives. The use of thermophilic microorganisms is one of many different ways that could increase the efficiency of industrial fermentations. Primarily through a large reduction in cooling costs, and the possibility of simultaneous saccharification and fermentation, in which (hemi)cellulose-hydrolysing enzymes, functioning optimally around 50 °C, can be used simultaneously with the fermentation process itself [2].

The strictly anaerobic *Pseudoclostridium thermosuccinogenes* is the only known thermophile to produce succinic acid as one of its major fermentation products (along with acetic acid, lactic acid, formic acid, ethanol, and hydrogen gas) [3, 4]. It is a close relative of the much better studied *Hungateiclostridium thermocellum* and *Hungateiclostridium cellulolyticum*, both efficient cellulose degraders that produce ethanol; *Pseudoclostridium thermosuccinogenes* is incapable of cellulose degradation, instead it is able to grow rapidly on inulin, a fructose polymer (as well as a range of C5 or C6 monosaccharides).

**Fig. 1:**
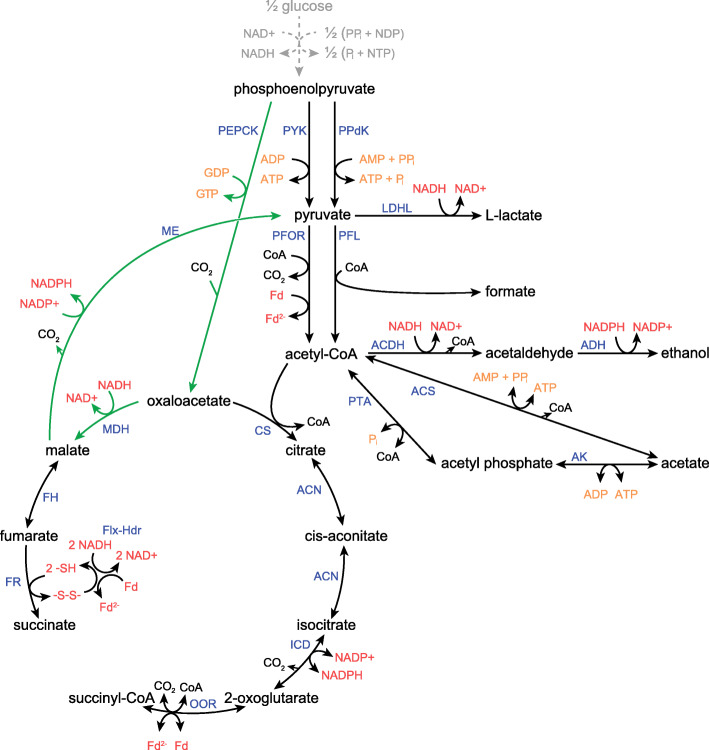
Metabolic pathways from phosphoenolpyruvate (PEP) to the different fermentation products in *P. thermosuccinogenes*. The dashed grey arrow represents the glycolysis, which relies on a PP_i_-dependent phosphofructokinase. The green arrows represent the malate shunt for the conversion of PEP to pyruvate. ACDH, acetaldehyde dehydrogenase; ACN, aconitase; ACS, acetyl-CoA synthetase; ADH, alcohol dehydrogenase; AK, acetate kinase; CS, citrate synthase; Flx-Hdr, NADH dehydrogenase/heterodisulfide reductase bifurcation complex; FH, fumarate hydratase; FR, fumarate reductase; ICD, isocitrate dehydrogenase; LDHL, l-lactate dehydrogenase; MDH, malate dehydrogenase; ME, malic enzyme; OOR, 2-oxoglutarate:ferredoxin oxidoreductase; PEPCK, phosphoenolpyruvate carboxykinase; PFL, pyruvate formate lyase; PFOR, pyruvate:ferredoxin oxidoreductase; PPdK, pyruvate, phosphate dikinase; PTA, phosphate acetyltransferase; PYK, pyruvate kinase. The figure is adapted from [4]

The metabolic pathway to succinic acid in *P. thermosuccinogenes* involves the fixation of a CO_2_ molecule by the GTP-dependent phosphoenolpyruvate carboxykinase (PEPCK), converting phosphoenolpyruvate (PEP) into oxaloacetate, while forming GTP from GDP [4]. Oxaloacetate is then converted into succinate via malate dehydrogenase, fumarate hydratase, and, finally, fumarate reductase (Fig. 1). The PEPCK reaction is known to operate close to its thermodynamic equilibrium, so it is likely that CO_2_ concentrations can impact the growth of *Pseudoclostridium thermosuccinogenes* and/or its production of succinic acid, as is the case with several other natural succinic acid producers, which are typically considered capnophiles (organisms that thrive in the presence of CO_2_) [5]. We previously speculated that the use of GTP, rather than ATP, for PEPCK and sugar phosphorylation might allow growth at lower CO_2_ concentrations by modulating the thermodynamics [6]. Other reactions in the central metabolism that could be affected by different CO_2_ concentrations include those catalyzed by malic enzyme (ME) and pyruvate ferredoxin oxidoreductase (PFOR), facilitating the oxidative decarboxylation of malate to pyruvate, and that of pyruvate to acetyl-CoA, respectively.

The aim of this study was to investigate how different CO_2_ concentrations affect the production of succinic acid and other fermentation products by *P. thermosuccinogenes*. Fermentations were carried out in bioreactors, directly comparing 20% sparged CO_2_ (v/v) with 1%, at which CO_2_ was found to become limiting. A transcriptome analysis was conducted in order to look further into the mechanisms behind the observed metabolic changes triggered by CO_2_ limitation.

## Results

**Fig. 2:**
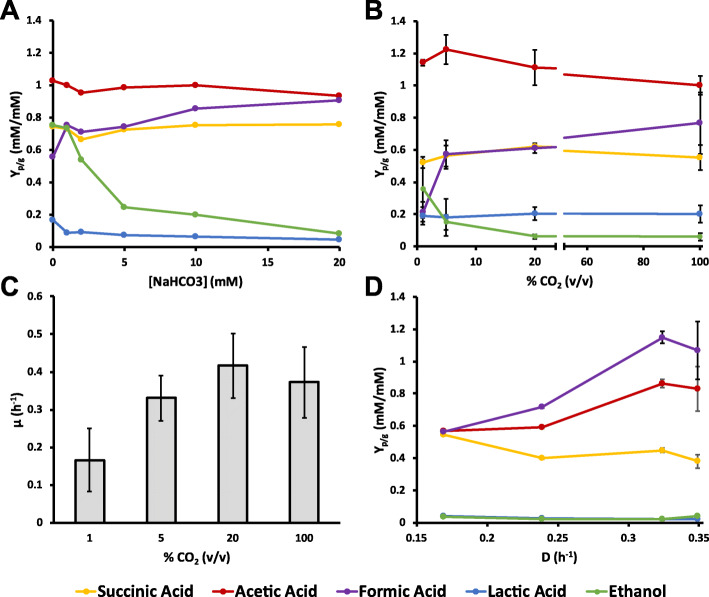
Testing the effect of available CO_2_. (**a**) Yield of moles fermentation product produced per consumed mole of glucose (starting concentration of 5 g/l) in serum bottle experiments containing different starting concentrations of NaHCO_3_. 1 ml of inoculum containing ~ 50 mM NaHCO_3_ was used for 50 ml medium, so the true NaHCO_3_ concentrations are ~ 1 mM higher. (**b**) Yield of moles fermentation product produced per consumed mole of glucose (starting concentration of 25 g/l) in 0.5 l batch fermentations with different concentrations of CO_2_ in N_2_ (v/v) sparged at 1 l/h. Every data point is the average of data from at least 3 independent fermentations, with the error bars representing the standard deviation. (**c**) Growth rates of the fermentations in B. (**d**) Yield of moles fermentation product produced per consumed mole of glucose (starting concentration of 5 g/l) in a continuous fermentation set a different (step-wise reduced) dilution rates. Every data point is the average of 3 measurements of the same steady state, taken at different time points, with the error bars representing the standard deviation

In order to investigate the effect of the available CO_2_ on the formation of succinic acid and other fermentation products by *P. thermosuccinogenes*, a series of bottle cultivations was conducted with medium containing different NaHCO_3_ concentrations. A range of NaHCO_3_ concentrations from 0 to 20 mM was tested, although the true concentrations are approximately 1 mM higher, through carry-over from the inoculum. 5 g/l of glucose was used, of which generally only little more than half was consumed, due to the rapid acidification of the medium. The results are presented in Fig. 2a and show a stark increase in ethanol yield at lower NaHCO_3_ concentrations, and a modest decrease in formic acid yield. Surprisingly, succinic acid did not show an apparent change in yield, and neither did acetic acid and lactic acid.

Following the small bottle experiment, a similar, but better-controlled, batch fermentation experiment was carried out using laboratory scale bioreactors, containing 0.5 l medium with 25 g/l glucose. 5 g/l yeast extract was added, instead of 1 g/l, which was otherwise found to be limiting (data not shown). Similar to the bottle experiments, 1 g/l of l-cysteine was used to reduce the medium and remove any traces of oxygen. Instead of NaHCO_3_ additions, the medium was sparged with different concentrations of CO_2_ in N_2_ at 1 l/h, while being stirred at 200 rpm. The pH was maintained at 7.0. The results of the batch fermentations are shown in Fig. 2b, and are comparable to what was observed for the bottle experiments, with ethanol yield increasing and formic acid yield decreasing at lower CO_2_ concentrations. Figure. 2c shows the growth rates at the different tested CO_2_ concentrations, which are reduced over two folds at 1%, compared to 20%. No growth was observed at all when the reactor was sparged with 100% N_2_.

To try to discriminate between direct effects of CO_2_ concentrations and indirect effects via differences in growth rates, a chemostat fermentation was conducted at different dillution rates and a fixed CO_2_ concentration. The chemostat was run similar to the batch fermentations, but medium with 5 g/l of glucose, 1 g/l yeast extract, and 0.5 g/l l-cysteine was used, and the medium was sparged with 50% CO_2_. The continuous dilution with fresh medium was initiated at the end of the exponential phase, starting with the highest dilution rate. Figure 2d shows the results of the continuous fermentation. Virtually no ethanol and lactic acid are produced during any of the tested dilution rates, suggesting that the previously observed increased ethanol yield was not the result of a lower growth rate, but is more directly the result of the lower CO_2_ concentration. Formic acid, on the other hand, is produced in large amounts, and its yield also appears to decrease with decreasing growth rate. Acetic acid appears to follow a similar trend, whereas succinic acid yield seems to increase slightly. As such, it seems that the formic acid yield change might indeed be the result of the lower growth rate. Nevertheless, the change in acetic acid (and succinic acid), which was not observed (as strongly) during the batch fermentations could also indicate that another, unaccounted mechanism might be behind the observation.

**Fig. 3:**
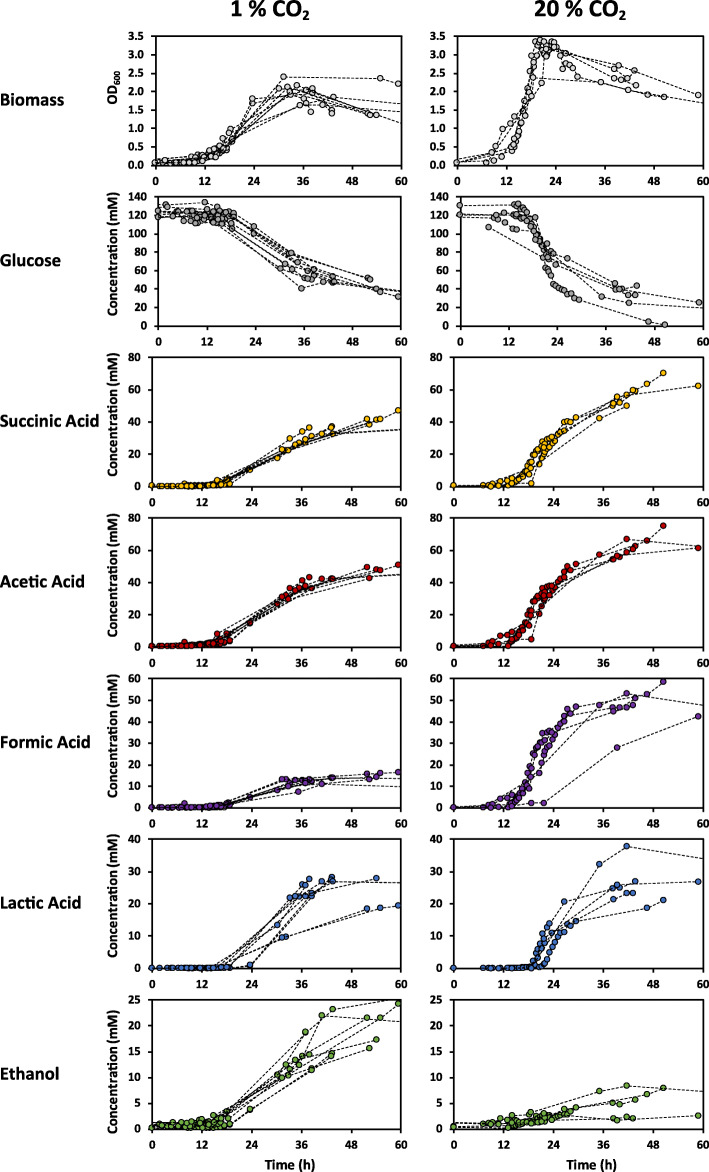
Fermentation data. Optical density, and concentrations of glucose and fermentation products over-time of a series of batch fermentations (0.5 l) sparged with either 1% or 20% CO_2_ in N_2_ (v/v) at 1 l/h. The dashed lines connect data points from the same run

**Fig. 4:**
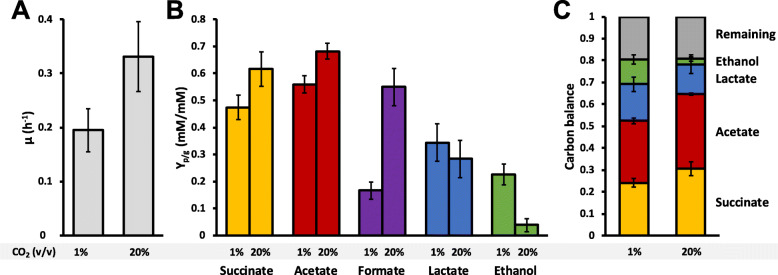
Overview of fermentation results. Growth rate (**a**), fermentation product yields on glucose (**b**), and carbon balance per mole of glucose (**c**) of the series of batch fermentations presented in Fig. 3. Error bars represent standard deviaitons. The carbon balance is calculated by converting glucose to C-moles (i.e. multiplied by 6) and multiplying succinate, acetate, lactate, and ethanol by 3

In order to look more closely at what might be behind the increase in ethanol yield and decrease in formic acid yield, another set of batch fermentations was carried out, directly comparing sparging with 1% versus 20% CO_2_. RNA samples were collected for sequencing to investigate transcriptional changes. The data of the fermentations are presented in Figs 3 and 4.

Figure 3 shows that glucose consumption rates drop near the end of the fermentations, and that not all of the 25 g/l of glucose is consumed. A disparity between 1 and 20% CO_2_ is also evident. The OD_600_ stabilizes within 24 h, after which it also shows a rapid decrease. This suggests that cells are dying and/or sporulating, which would also explain the decrease in glucose consumption, and is presumably caused by salt stress resulting from titration with KOH [7, 8]. Lactate production starts at the transition to the stationary phase. It was further noted that at 1% CO_2_ the cells were elongated – a typical stress response [9, 10] – and that the cells formed much more sticky or slimy cell-pellets, suggestive of an increased presence of extracellular polysaccharides – another common stress response [11, 12].

As before, the results show a stark decrease in formate yield, which dropped from 0.53 to 0.17 mM per mM glucose during CO_2_ limitation, while ethanol yield increased from 0.05 to 0.23 mM. Lactate yield also increased slightly (0.29 to 0.34 mM/mM), and succinate and acetate yields decreased slightly (0.64 to 0.47 and 0.69 to 0.56 mM/mM, respectively). Overall, the amount of glucose channelled to succinate, acetate, lactate and ethanol combined remained identical during both conditions, as can be seen in Fig. 4c. Approximately 20% of glucose is unaccounted for, and is at least partly represented by the formed biomass.

**Fig. 5:**
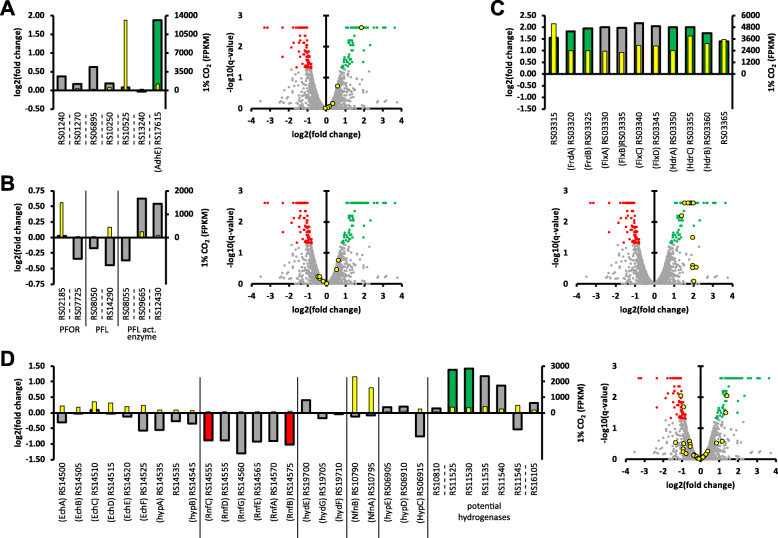
Selected results from differential expression analysis. Positive fold change represents an increase of mRNA coverage during CO_2_ limitation (i.e. 1% versus 20% CO_2_). In the volcano plots, green data points depict genes with a statistically significant increase (i.e. q-value < 0.05) during CO_2_ limitation and red data points those with a statistically significant decrease in coverage during CO_2_ limitation. Yellow data points correspond to the genes presented in the accompanying bar graph. In the bar graphs, the wide bars display the log2(fold change) and the narrow (yellow) bars display the mean coverage in FPKM during 1% CO_2_. **a** Results for potential alcohol dehydrogenases (including AdhE). **b** Results for two annotated pyruvate:ferredoxin oxidoreductases (PFOR), two annotated pyruvate formate lyases (PFL), and three (potential) PFL-activating enzymes. **c** Results for the ‘succinate operon’, harbouring fumarate hydratase (CDQ83_RS03365), an electron bifurcating NADH dehydrogenase/heterodisulfide reductase complex (CDQ83_RS03360–30), fumarate reductase (CDQ83_RS03325–20), and a hypothetical protein (CDQ83_RS03315; potentially a succinic acid transporter). **d** Results for the energy-converting [NiFe] hydrogenase with hydrogenase maturation factors (Ech; CDQ83_RS14500–45); the ion-translocating reduced ferredoxin: NAD^+^ oxidoreductase (Rnf; CDQ83_RS14555–75); HydEFG [FeFe] hydrogenase maturation factors; electron bifurcating ferredoxin: NADP^+^ reductase (Nfn; CDQ83_RS10790–95); HypCDE [NiFe] hydrogenase maturation factors (CDQ83_RS06905–15); and three potential hydrogenases (CDQ83_RS10810, CDQ83_RS11525–45, and CDQ83_RS16105)

A differential expression analysis was performed through RNA sequencing, in order to look further into mechanisms that could be behind the observed effects. Biological triplicates of exponentially growing cells from the batch fermentations were used for RNA sequencing. 5.4–10.4 million reads (paired-end, 150 bp) were generated per sample and used to conduct a differential expression analysis between 1 and 20% CO_2_ (Supplementary data 1). As can be seen in Fig. 5a, one of the seven (putative) alcohol dehydrogenases annotated in *P. thermosuccinogenes* is significantly overexpressed during CO_2_ limitation, namely *adhE* (CDQ83_RS17615) – a bifunctional acetaldehyde-CoA/alcohol dehydrogenase that was previously shown to be the relevant isoform for ethanol formation in *H. thermocellum* and *T. saccharolyticum* [13]. Surprisingly, neither of the two annotated PFOR genes were differentially expressed, as shown in Fig. 5b, nor were any of the genes coding for other central metabolic enzymes involving CO_2_ formation or fixation. None of the genes for PFLs or (putative) PFL-activating enzymes were differentially expressed either (Fig. 5b).

**Fig. 6:**
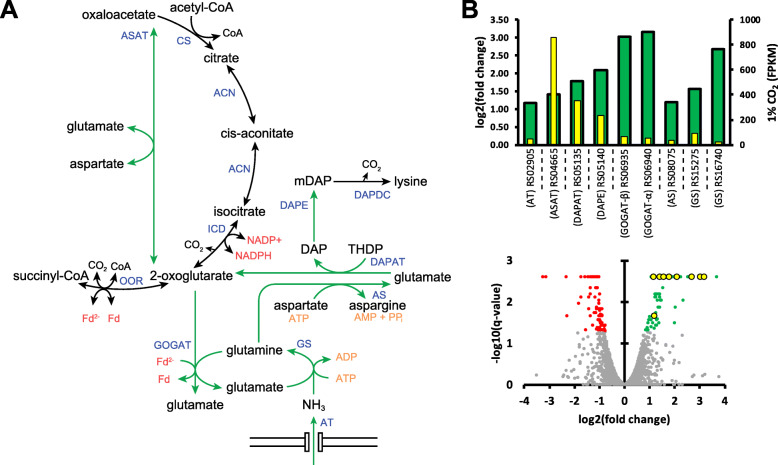
**a** Overview of the statistically significantly overexpressed reaction (green) involved in glutamate metabolism during CO_2_ limitation. ACN, aconitase; AS, aspargine synthase (CDQ83_RS08075); ASAT, aspartate transaminase (CDQ83_RS04665); AT, ammonium transporter (CDQ83_RS02905); CS, citrate synthase; DAPAT, l,l-diaminopimelate aminotransferase (CDQ83_RS05135); DAPDC, diaminopimelate decarboxylase; DAPE, diaminopimelate epimerase (CDQ83_RS05140); GOGAT, ferredoxin-dependent glutamate synthase (CDQ83_RS06935–40); GS, glutamine synthethase (CDQ83_RS15275 & CDQ83_RS16740); ICD, isocitrate dehydrogenase; OOR, 2-oxoglutarate:ferredoxin oxidoreductase. **b** Results from differential expression analysis for the genes involved in the highlighted reactions in A. Positive fold change represents an increase of mRNA coverage during CO_2_ limitation (i.e. 1% versus 20% CO_2_). In the volcano plots, green data points depict genes with a statistically significant increase (i.e. q-value < 0.05) during CO_2_ limitation and red data points those with a statistically significant decrease in coverage during CO_2_ limitation. Yellow data points correspond to the genes presented in the accompanying bar graph. In the bar graphs, the wide bars display the log2(fold change) and the narrow (yellow) bars display the mean coverage in FPKM during 1% CO_2_

The most differentially over-expressed genes during CO_2_ limitation include a glutamate synthase (CDQ83_RS06935 and CDQ83_RS06940), glutamine synthetase (CDQ83_RS16740), as well as several other genes related to glutamate/amino acid metabolism, summarized in Fig. 6. Furthermore, three genes (CDQ83_RS11600, CDQ83_RS11605, and CDQ83_RS11610) that seem to encode for a putative oxidoreductase complex are highly over-expressed during CO_2_ limitation as well. CDQ83_RS11600 encodes a small DUF1667 domain-containing protein; CDQ83_RS11605 an FAD-dependent oxidoreductase related to the small chain of thioredoxin reductase, glutamate synthase, CoA disulfide reductase, and ferredoxin-NADP^+^ reductase; and CDQ83_RS11610 an NAD(P)/FAD-dependent oxidoreductase related to L-2-hydroxyglutarate dehydrogenase (mitochondrial) and glycerol-3-phosphate dehydrogenase. The three genes are located downstream of a glycerol-3-phosphate responsive antiterminator protein and upstream of a glycerol kinase.

**Fig. 7:**
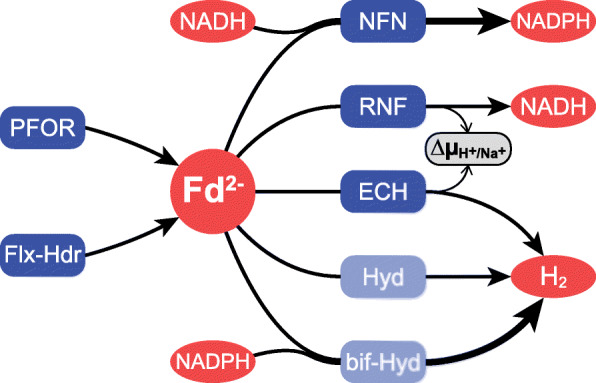
Overview of major (trans) hydrogenase reactions involving ferredoxin in *P. thermosuccinogenes*. Arrow represent the flow of electrons. PFOR: pyruvate:ferredoxin oxidoreductase; Flx-Hdr: NADH dehydrogenase/heterodisulfide reductase bifurcation complex; NFN: NADH-dependent reduced ferredoxin: NADP^+^ oxidoreductase; RNF: ion-translocating reduced ferredoxin: NAD^+^ oxidoreductase; ECH: energy-converting [NiFe] hydrogenase; Hyd: hydrogenase; bif-Hyd: bifurcating hydrogenase. Differential expression of the corresponding genes (and their locus tags) are presented in Fig. 5. It is not entirely clear what hydrogenases are present besides ECH. The presence of several genes potentially encoding hydrogenases, and of [FeFe] hydrogenase maturation factors does suggest *P. thermosuccinogenes* possesses additional hydrogenases

The ‘succinate operon’, encoding fumarate hydratase, fumarate reductase and the NADH dehydrogenase/heterodisulfide reductase bifurcation complex showed a statistically significant increase in expression level of approximately 4-fold (Fig. 5c). Conversely, the ion-translocating reduced ferredoxin: NAD^+^ oxidoreductase (RNF) complex showed a decrease of approximately 2-fold. Besides the RNF complex, *P. thermosuccinogenes* possesses several other known enzymes involved in (trans) hydrogenation, summarized in Fig. 7; none of these are differentially expressed, except for a cluster of genes related to NADH-quinone oxidoreductase (CDQ83_RS11525–45) that could potentially encode a hydrogenase complex (Fig. 5d).

Interestingly, a large cluster of genes (CDQ83_RS11365–420 that appear to be organized into three adjacent operons) predicted to be involved in fucose and mannose metabolism is overexpressed during CO_2_ limitation. It is plausible that this relates to the sticky phenotype that was observed for the cells grown at 1% CO_2_.

## Discussion

### Cofactor fluxes

Figure 1 shows an overview of the pathways towards the different fermentation products. The yields of those products, together with their associated cofactor stoichiometry, starting from PEP, were used to calculate the cofactor (re) generation at 1 and 20% CO_2_. The results of the flux balance calculations (Supplementary file 2) are presented in Table 1. During 1% CO_2_, 0.42 mol extra ferredoxin per mole glucose was reduced by PFOR, resulting from the decreased pyruvate formate lyase (PFL) flux. Assuming that fumarate reductase is indeed linked to ferredoxin reduction (through NADH dehydrogenase/heterodisulfide reductase complex, as proposed previously [14, 15]), it follows that overall, 0.26 mol extra ferredoxin is reduced during CO_2_ limitation. Simultaneously, 0.25 mol fewer NADH and 0.18 mol extra NADPH is oxidized (the latter through the NADPH-dependent alcohol dehydrogenase of *P. thermosuccinogenes* [16]), assuming the transhydrogenase activity of the malate shunt has not changed [17]. In total, this would constitute a relative redox surplus of 0.33 electron pairs during CO_2_ limitation. Presumably, this leads to extra H_2_ production. Unfortunately, no H_2_ data is available from the bioreactor fermentations. Serum bottle experiments, identical to those presented in Fig. 2a did show an increase in H_2_ production from 0.22 mol H_2_ per mole glucose at 20 mM NaHCO_3_ to 0.44 mol H_2_ at 1 mM NaHCO_3_, suggesting indeed that more H_2_ is formed during CO_2_ limitation (data not shown). If we simply assume that 1 ATP-equivalent is generated per PEP to pyruvate conversion, 0.18 mol fewer ATP is generated during CO_2_ limitation, of which the majority (0.13 mol) is due to the decrease in acetate yield.

**Table 1:** Changes in cofactor fluxes during CO_2_ limitation. Moles of different cofactors or pathway intermediates generated per mole of glucose consumed, calculated using the different product yields as shown in Fig. 4 and stoichiometry of the pathways from phosphoenolpyruvate to the different fermentation products (i.e. the NADH/ATP-equivalents consumed/produced in glycolysis are not considered), as depicted in Fig. 1. Calculations based on the assumption that there is no difference in transhydrogenation by the malate shunt between 1 and 20% CO_2_, and that 1 ATP-equivalent is formed in the conversion from phosphoenolpyruvate to pyruvate (Additional file 2)

	1% CO_2_	20% CO_2_	Difference
Acetyl-CoA	0.79	0.73	0.05
Fd_reduced_ (PFOR only)	0.62	0.20	0.42
Fd_reduced_	1.09	0.84	0.26
NADH	−1.99	−2.25	0.25
NADPH	−0.23	−0.05	−0.18
Total redox cofactors	−1.13	−1.45	0.33
ATP (acetate kinase only)	0.56	0.68	−0.13
ATP-equivalents	2.16	2.34	−0.18

Following these results, it seems that during CO_2_ limitation mechanisms must exist for the oxidation of the extra reduced ferredoxin and NADH, as well as for the reduction of the extra NADP^+^, which strongly suggests that (besides increased hydrogen production) electrons are being transferred from ferredoxin and/or NADH to NADP^+^.

### PFOR versus PFL

Upon CO_2_ limitation, decarboxylation reactions (where a carboxyl group is removed) become thermodynamically more favourable, whereas the reverse (i.e. carboxylation) becomes less favourable [18]. A large number of metabolic pathways, both catabolic and anabolic, involve (de) carboxylation reactions and it is therefore likely that the observed effects during CO_2_ limitation are either directly or indirectly related to the thermodynamic changes. In general, a certain amount of CO_2_ is required for many microorganisms to thrive [19, 20], as was also demonstrated by the complete lack of growth of *P. thermosuccinogenes* during fermentations sparged with pure N_2_. The predominant catabolic (de) carboxylation reactions of *P. thermosuccinogenes* are PEPCK, malic enzyme, and PFOR. Phosphogluconate dehydrogenase in the oxidative pentose phosphate pathway is only marginally expressed, while isocitrate dehydrogenase and 2-oxoglutarate:ferredoxin oxidoreductase only fulfil an anabolic role (due to the incomplete TCA-cycle).

Of the three major catabolic (de) carboxylases, only PEPCK operated in the CO_2_-fixing direction, through which the production of succinic acid leads to net CO_2_ fixation, while the malate shunt does not lead to net CO_2_ fixation (or generation), because of subsequent decarboxylation by malic enzyme. In fact, the amount of CO_2_ required by PEPCK for the observed succinic acid production at 1% CO_2_ (~ 20 mmol in a 24 h window) is higher than the CO_2_ provided (0.01 l/h * 24 h * 22.4^− 1^ mol/l = ~ 11 mmol), which can only mean that endogenous CO_2_ is being used to facilitate succinic acid formation. The source of endogenous CO_2_ is PFOR (being the only anabolic net-CO_2_-forming reaction), which produces 0.62 mol CO_2_ per mole glucose, compared to the 0.47 mol required for succinic acid production. During 20% CO_2_, only 0.20 mol CO_2_ is produced by PFOR versus 0.64 mol required for succinic acid. Therefore, it seems that the switch from PFL to PFOR (i.e. from formate production to that of CO_2_ and reduced ferredoxin) allows succinic acid formation and its concomitant NADH sink to proceed in the absence of the required exogenous CO_2_. The re-oxidation of more than one NADH through succinate production allows the subsequent generation of extra ATP via the (redox neutral) pathway to acetate.

Whether the switch from PFL to PFOR is intentional (i.e. regulated) is not clear. It does not appear to be regulated on a transcriptional level. (Post)-Translational or allosteric regulation is still possible, but it could simply be a thermodynamic effect, since PFOR becomes more favourable relative to PFL during CO_2_ limitation. Recent data from Dash et al. (2019) show that the free energy change of PFOR in *Hungateiclostridium thermocellum* (a close relative of *P. thermosuccinogenes*) changes quite drastically during the course of a batch fermentation, being close to 0 in the beginning of the fermentation [21]. With a ΔG allowed close to 0, at which its rate is very sensitive to changes in reactant concentrations, it is fully plausible that the switch from PFL to PFOR in *P. thermosuccinogenes* upon CO_2_ limitation is a thermodynamic effect.

### Changes in redox metabolism

The overall consequence of the observed metabolic changes is an increased need for ferredoxin and NADH oxidation, and NADP^+^ reduction. It is credible then to assume that part of the ferredoxin and NADH are used for the reduction of NADP^+^, which could occur through the electron bifurcating ferredoxin: NADP+ reductase (NFN; Fig. 7) [22]. Again, no transcriptional changes occur that could indicate increased NFN activity. Of course, it is still wholly plausible that there is an increase in NFN-flux. The flux through the malate shunt might also change, increasing NADH to NADPH transhydrogenase activity. Alternatively, the putative oxidoreductase complex that is most differentially upregulated might encode a novel protein complex involved in the transfer of electrons from ferredoxin (and NAHD) to NADP^+^. Further research into this putative oxidoreductase complex is required, however. Its genomic association with glycerol kinase would in fact suggest a relation to membrane lipid synthesis.

Unlike NFN, the ion-translocating reduced ferredoxin: NAD^+^ oxidoreductase (RNF) seems to be transcriptionally downregulated, albeit statistically significant for only two of the six subunits. Conversely, the succinic acid operon, harbouring the bifurcating NADH dehydrogenase/heterodisulfide reductase complex (Flx-Hdr), is upregulated, albeit also not statistically significant for all subunits. RNF transfers electrons from ferredoxin to NAD^+^, whereas Flx-Hdr transfers electrons from NADH to ferredoxin (and thiols) [14, 15]. The transcriptional effects could therefore indicate that the excess of NADH is more pressing than the excess of ferredoxin, as they would result in reduced transfer of electrons from ferredoxin to NADH. This is conceivable, considering the fact that ferredoxin can be re-oxidized relatively easily through the formation of hydrogen. The observed stress could therefore be the result of a decreased NAD^+^/NADH ratio, which is (for example) known to inhibit glyceraldehyde-3-phosphate dehydrogenase, decreasing glucose consumption and growth [23–25].

Sridhar & Eiteman (2001) attempted to decrease the NAD^+^/NADH ratio of *P. thermosuccinogenes* through the addition of 85% H_2_ to the headspace, which resulted in almost completely abolished succinate and formate production and greatly reduced lactic acid production, with their fluxes completely diverted to ethanol [26]. This is surprising, as in most natural succinate producers H_2_ addition enhances succinate production [27–29]. Nevertheless, it fits the notion that *P. thermosuccinogenes* has a unique fumarate reductase, coupling succinate formation to ferredoxin reduction [15], since inhibition of hydrogenases by high H_2_ partial pressure would impair ferredoxin reducing reactions [30, 31]. In the same study, confusingly, decreased culture redox potential (CRP; assumed to increase NADH availability) by addition of Na_2_S resulted in the complete opposite effect as H_2_ addition. Overall, there seems to be a very complex interplay between pH, CRP, as well as H_2_ and CO_2_ on the fermentation profile of *P. thermosuccinogenes*.

### CO_2_ limitation in succinic acid producers

The premise of this investigation was the idea that succinic acid formation would be strongly impacted by the concentration of CO_2_, asserted from the CO_2_-fixing PEPCK reaction, which is believed to operate close to equilibrium [32], as well as from evidence from other succinic acid producers. A decrease of 26% in succinate yield was observed, which albeit significant is smaller than the 65% decrease seen for *Anaerobiospirillum succiniciproducens*, and 72% for *Actinobacillus succinogenes*, summarized in Table 2 with results from literature. *Mannheimia succiniciproducens* shows a decrease of 14%. However, the lowest amount of CO_2_ tolerated by *M. succiniciproducens* was many times higher than that for *P. thermosuccinogenes*. Beyond the general observed decrease in succinic acid yield upon CO_2_ limitation, the response in terms of the other fermentation products varies widely between different succinic acid producers (Table 2). E.g. where *P. thermosuccinogenes* shows a stark decrease in formate yield, *A. succinogenes* shows an increase, while the formate yield of *A. succiniproducens* is unaffected, but was already low to begin with. In more recent work into CO_2_ limitation in *A. succinogenes*, two specific CO_2_ threshold concentrations were found. Below 8.4 mM dissolved CO_2_ (or 37% saturation), the glucose consumption rate decreased while the flux distribution (i.e. yields) between succinate, acetate and formate remained constant. Below 3.9 mM dissolved CO_2_ (or 17% saturation), the yield of succinate also decreased, as flux was diverted towards other products [38].

**Table 2:** Comparison with other studies. Studies showing the effects of CO_2_ limitation on succinic acid producers grown on glucose, in terms of growth and product yields. SA: succinate, AA: acetate, FA: formate, LA: lactate, EtOH: ethanol, PA: pyruvate. Biomass and product yields are given in mol/mol glucose. Growth rate in h^−1^

Organism	SA	AA	FA	LA (PA)	EtOH	Biomass	Growth rate	CO_2_ provided	Ref.
*Anaerobiospirillum succiniciproducens*^a^	1.21	0.67	0.12	0.00	0.00	1.13	?	0.98 mol MgCO_3_/mol glucose	[33]
	0.43	0.16	0.12	0.87	0.09	0.44	?	0.065 mol MgCO_3_/mol glucose	
*Actinobacillus succinogenes*	0.69	0.84	0.88	?	0.18	1.07	?	1.00 mol MgCO_3_/mol glucose	[28]
	0.19	0.81	1.20	?	0.71	1.08	?	0.10 mol MgCO_3_/mol glucose	
*Mannheimia succiniciproducens*^b^	0.70	?	?	?	?	0.18 g/g	1.12	100% CO_2_ at 0.25 vvm (23 mM)	[34]
	0.60	?	?	?	?	0.13 g/g	0.78	37% CO_2_ at 0.25 vvm (8.7 mM)	
*Escherichia coli* AFP111^c^	1.10	0.12	?	(0.20)	0.12	?	?	50% CO_2_ at 0.42 vvm	[35]
	0.79	0.09	?	(0.39)	0.08	?	?	3% CO_2_ at 0.42 vvm	
*Escherichia coli* BA207^d^	1.46	0.48	?	(0.04)	?	1.72 g/l	?	100% CO_2_ at 0.13 vvm	[36]
	1.07	0.27	?	(0.12)	?	0.42 g/l	?	0% CO_2_ at 0.13 vvm	
*Corynebacterium glutamicum*^e^	0.87	0.28	?	0.42	?	–	0	100% CO_2_	[37]
	0.29	0.13	?	1.22	?	–	0	0% CO_2_	
*Pseudoclostridium thermosuccinogenes*	0.64	0.69	0.53	0.29	0.05	?	0.33	20% CO_2_ at 0.033 vvm	This study
	0.47	0.56	0.17	0.35	0.23	?	0.20	1% CO_2_ at 0.033 vvm	

^a^Growth rate decreased by almost 50% during CO_2_ limitation

^b^*M. succiniproducens* shows severely suppressed growth at CO_2_ concentrations below 8.7 mM

^c^*Escherichia coli* AFP111 has mutations in pfl, ldhA, ptsG. During 0% CO_2_ succinic acid yield dropped to 0.06

^d^*Escherichia coli* BA207: *E. coli* K12, ΔpflB, ΔldhA, Δppc, pTrc-pncB-pyc

^e^Non-growing high-density cell suspensions

Like *P. thermosuccinogenes*, *A. succiniciproducens* has a PFOR and a PFL [33], while *A. succinogenes* and *M. succiniciproducens* have a pyruvate dehydrogenase (PDH) instead of a PFOR [39, 40], forming NADH instead of reduced ferredoxin. Additionally, *P. thermosuccinogenes* has a GTP-dependent PEPCK, whereas the other three have ATP-dependent versions. We previously speculated that GTP-dependent PEPCK might allow growth at lower CO_2_ concentrations [6]. However, the data from literature presented in Table 2 are not sufficient to support that, as not enough other succinate producers relying on GTP-dependent PEPCK (e.g. *Fibrobacter succinogenes* [41]) have been studied during CO_2_ limitation and the tested limiting CO_2_ concentrations cannot be adequately compared.

## Conclusions

Through bioreactor cultivations of *Pseudoclostridium thermosuccinogenes* sparged with 1 and 20% CO_2_ we studied the effect of CO_2_ limitation on its metabolism. Formate yield is greatly reduced as the pyruvate to acetyl-CoA flux shifts from pyruvate formate lyase (PFL) to pyruvate:ferredoxin oxidoreductase (PFOR). This shift is presumably caused by more favourable decarboxylase thermodynamics of PFOR upon CO_2_ limitation, but might also be actively regulated, as the resulting endogenous CO_2_ formation is able to compensate the CO_2_ required to sustain the succinate flux. Succinate yield is (only) reduced by 26%. Acetate yield is slightly reduced as well, while that of lactate is slightly increased. CO_2_ limitation also prompts the formation of significant amounts of ethanol, which is only marginally produced during CO_2_ excess. Overall, the changes in those product yields are associated with increased ferredoxin and NAD+ reduction, and increased NADPH oxidation during CO_2_ limitation, which must result in altered (trans) hydrogenation mechanisms of those cofactors, in order to keep them balanced. Transcriptional changes show a clear overexpression of an alcohol dehydrogenase (*adhE*), while no change in PFL and PFOR expression is observed. Transcription results are more ambiguous regarding the altered (trans) hydrogenation mechanisms, but they hint at a decreased NAD^+^/NADH ratio, which might ultimately be responsible for the stress observed during CO_2_ limitation.

## Methods

### Medium composition and bottle cultivations

*P. thermosuccinogenes* DSM 5809 was routinely cultivated anaerobically in 120-ml serum bottles containing 50 ml medium, incubated at 60 °C.

Adapted bicarbonate-buffered CP medium was used that contained per liter 0.408 g KH_2_PO_4_, 0.534 g Na_2_HPO_4_·2H_2_O, 0.3 g NH_4_Cl, 0.3 g NaCl, 0.1 g MgCl_2_·6H_2_O, 0.11 g CaCl_2_·2H_2_O, 4.0 g NaHCO_3_, 0.1 g Na_2_SO_4_, 1.0 g l-cysteine, 1.0 g yeast extract (BD Bacto), 0.5 mg resazurin, 1 ml vitamin solution, 1 ml trace elements solution I, and 1 ml trace elements solution II [4, 42]. The medium was autoclaved in serum bottles under 80:20 N_2_/CO_2_ atmosphere with ∼70 kPa overpressure, containing a final volume of 50 ml medium. A solution containing NaHCO_3_ and l-cysteine was autoclaved separately and added later as well as a solution containing CaCl_2_·2H_2_O, to which the vitamin solution was added after it was autoclaved. Glucose was also autoclaved separately and added later to a final concentration of 2 g/l or 5 g/l.

The vitamin solution, which was 1000× concentrated, contained per liter 20 mg biotin, 20 mg folic acid, 100 mg pyridoxine-HCl, 50 mg thiamine-HCl, 50 mg riboflavin, 50 mg nicotinic acid, 50 mg Ca-d-pantothenate, 1 mg vitamin B_12_, 50 mg 4-aminobenzoid acid, and 50 mg lipoic acid. Trace elements solution I, which was 1000× concentrated, contained per liter 50 mM HCl, 61.8 mg H_3_BO_4_, 99.0 mg MnCl_2_·4H_2_O, 1.49 g FeCl_2_·4H_2_O, 119 mg CoCl_2_·6H_2_O, 23.8 mg NiCl_2_·6H_2_O, 68.2 mg ZnCl_2_, and 17.0 mg CuCl_2_·2H_2_O. Trace elements solution II, which was 1000× concentrated, contained per liter 10 mM NaOH, 17.3 mg Na_2_SeO_3_, 33.0 mg Na_2_WO_4_·2H_2_O, and 24.2 mg Na_2_MoO_4_·2H_2_O.

To test the effect of the different NaHCO_3_ concentrations in bottle cultivations, the medium was buffered with 10 g/l MOPS instead, and bottles were prepared under a 100% N_2_ atmosphere. The pH of the medium placed on ice had been set at 8.0, such that the pH at 60 °C would be ~ 7.4. NaHCO_3_ and l-cysteine were added after autoclaving from separate stock solutions, to their desired concentrations.

### Batch fermentations

Batch fermentations were carried out in DASGIP® BioBlock reactors (Eppendorf) with 0.5 l medium, that contained 5 g/l yeast extract and 25 g/l glucose. Bioreactors were autoclaved containing 420 ml of CP medium, lacking glucose, L-cysteine, NaHCO_3_, and CaCl_2_ with vitamins. After autoclaving, 50 ml glucose (250 g/l), 25 ml L-cysteine (20 g/l) and 5 ml CaCl_2_ (11 g/l) + vitamins were added. After the medium was fully reduced, inoculation was done using 1 ml overnight culture from a 120-ml serum bottle. The reactors were sparged with varying concentrations of CO_2_ in N_2_ at a rate of 1 l/h. The pH of the medium was kept at 7.0 by titration with 3 M KOH. The temperature was controlled at 60 °C and stirring was done at 200 rpm.

Fermentations were carried out over a period of approximately 3 days. Samples were taken over time to measure optical density, cell dry weight, and metabolite concentrations by HPLC.

### Chemostat fermentation

The continuous, chemostat fermentation was carried out in the same set-up as the batch fermentations. Glucose was intended to be the limiting component, therefore 5 g/l was used together with 1 g/l yeast extract. Furthermore, the l-cysteine concentration was halved to 0.5 g/l. The reactor was sparged with 50% (v/v) CO_2_ in N_2_, and the pH of the medium was kept at 7.0 by titration with 3 M KOH. The temperature was controlled at 60 °C and stirring was done at 200 rpm.

After inoculation, the culture was grown under batch conditions up to an OD_600_ of 0.8–1, after which the continuous feeding was started. The volume was kept constant by setting the outflow tube at the height corresponding to 0.5 l. The chemostat series at different dilution rates was started from the highest dilution rate, and steady states were assumed after three times the hydraulic retention time. Samples were taken from the outflow to measure optical density, cell dry weight, metabolite concentrations by HPLC and H_2_ concentrations by gas chromatography.

### HPLC

Glucose and fermentation products were analysed by HPLC using a Unity Lab Services ICS 5000+ system equipped with an Aminex HPX-87H column. The mobile phase contained 8 mM H_2_SO_4_ and was pumped at 0.8 ml/min through the column, which was kept at 60 °C. Samples and standards were prepared by mixing 160 μl with 40 μl of 10 mM DMSO internal standard in 5 mM H_2_SO_4_, in a 96-wells plate.

### RNA extraction and transcriptomics

15–45 ml samples were taken during the middle exponential growth phase in batch fermentations for transcriptome analysis. Samples were directly placed on ice, after which they were centrifuged for 10 min at 4800×g at 4 °C. The supernatant was removed and the pellet was resuspended in 10 ml RNA*later* Stabilization Solution (Qiagen) to inactivate RNases and stabilize the RNA. Samples were stored at 4 °C overnight and then transferred to − 20 °C until further processing.

To extract the RNA, 5 ml of the cell suspension was centrifuged for 15 min at 4800×g at 4 °C. All traces of the RNA*later* were removed and the pellet was resuspended in 0.5 mL of ice-cold TE buffer (pH 8.0). The samples were divided into two 2-ml screw-cap tubes containing 0.5 g zirconium beads, 30 μl 10% SDS, 30 μl 3 M sodium acetate (pH 5.2), and 500 μl water-saturated phenol, chloroform, and isoamyl alcohol at a ratio of 25:24:1 (pH 4.5 to 5) (Roti-Aqua-P/C/I; Carl Roth, Germany). Cells were disrupted in a FastPrep apparatus (MP Biomedicals) at 6000 rpm for 40 s. The tubes were centrifuged for 10 min at 10,000×g at 4 °C and the aqueous phase from both tubes was pooled in a new tube. 400 μL of chloroform was added to the samples, which were then centrifuged for 3 min at 21,000×g at 4 °C. 300 μL of the aqueous phase was finally transferred to a new tube, from which the RNA was purified using High Pure RNA Isolation Kit (Roche). RNA was eluted with 50 μL ultra-pure water and then stored at − 80 °C.

RNA integrity was verified using the Qsep100™ capillary gel electrophoresis system (BiOptic, Taiwan). Further quality control, rRNA depletion, library preparation, sequencing, and data analysis was carried out by BaseClear (The Netherlands). rRNA depletion was done using the MICROB*Express*™ Bacterial mRNA Enrichment Kit. Paired-end reads were generated with the Illumina NovaSeq 6000 system. Tophat2 version 2.1.1 was used to align the reads to the reference genome (NZ_NIOI01000002.1) with short read aligner Bowtie version 2.2.6 [43, 44]. Cufflinks version 2.2.1 was used to conduct the differential expression analysis [45].

## Supplementary information

**Additional file 1 : Supplementary file 1. Results of differential expression analysis.** Between CO_2_ limitation (i.e. group1) and CO_2_ excess (i.e. group2), using RNA samples of exponentially growing cells in biological triplicates.

**Additional file 2 : Supplementary file 2. Cofactor flux analysis.** Calculations of the cofactor flux analysis, based on the product yields obtained from the batch fermentations.

## Data Availability

All data generated or analysed during this study are included in this published article and its supplementary information files.

## Abbreviations

Flx-Hdr: Bifurcating NADH dehydrogenase/heterodisulfide reductase complex
ME: Malic enzyme
NFN: Electron bifurcating ferredoxin: NADP+ reductase
PEP: Phosphoenolpyruvate
PEPCK: Phosphoenolpyruvate carboxykinase
PFL: Pyruvate formate lyase
PFOR: Pyruvate ferredoxin oxidoreductase
RNF: Ion-translocating reduced ferredoxin: NAD^+^ oxidoreductase
