# Supplementary material for: Effects of CO2 limitation on the metabolism of Pseudoclostridium thermosuccinogenes
Source: BMC Microbiol. 2020 Jun 8;20:149. doi: 10.1186/s12866-020-01835-2 (PMC7282089; doi:10.1186/s12866-020-01835-2)
Supplement: Supplementary file 2 — Additional file 2 : Supplementary file 2. Cofactor flux analysis. Calculations of the cofactor flux analysis, based on the product yields obtained from the batch fermentations. [file 12866_2020_1835_MOESM2_ESM.docx]

# Supplementary data 1: Cofactor flux analysis

**Yields (mol/mol glucose)**

|  | 1% | St dev | 20% | St dev |
| --- | --- | --- | --- | --- |
| Acetate | 0.560019 | 0.032931 | 0.686697 | 0.029221 |
| Succinate | 0.474664 | 0.046133 | 0.637693 | 0.054757 |
| Lactate | 0.344226 | 0.069586 | 0.285975 | 0.066152 |
| Formate | 0.166577 | 0.031695 | 0.533579 | 0.056401 |
| Ethanol | 0.225331 | 0.039411 | 0.046433 | 0.024449 |

**Analysed system**

Assumed that:

- 1 ATP (equivalent) is generated per PEP to pyruvate conversion, regardless if pyruvate kinase; pyruvate, phosphate dikinase; or the malate shunt is used.
- There is no difference in the transhydrogenase activity of the malate shunt.
- fumarate reductase is linked to the electron bifurcating NADH dehydrogenase/heterodisulfide reductase complex.


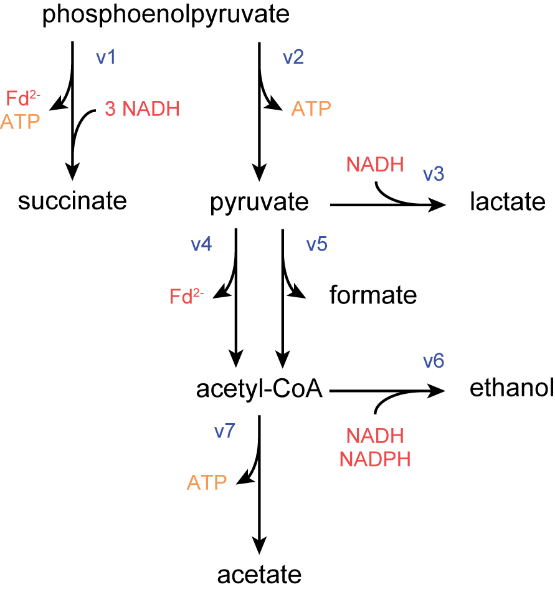


v1 = Y_SA/G_

v2 = v3 + v6 + v7 = Y_LA/G_ + Y_EtOH/G_ + Y_AC/G_

v3 = Y_LA/G_

v4 = v6 + v7 – v5 = Y_EtOH/G_ + Y_AC/G_ - Y_FA/G_

v5 = Y_FA/G_

v6 = Y_EtOH/G_

v7 = Y_AC/G_

**Cofactors/pathway intermediates generated (mol/mol glucose)**

|  |  | 1% CO_2_ | 20% CO_2_ |
| --- | --- | --- | --- |
| NADH | (-3 * v1) + (-1 * v3) + (-1 * v6) | -1.993549664 | -2.24548692 |
| NADPH | (-1 * v6) | -0.225330504 | -0.046433034 |
| Fd^2-^ | (1 * v1) + (1 * v4) | 1.093437725 | 0.837244389 |
| Fd^2-^ (v4 only) | (1 * v4) | 0.618773324 | 0.199551296 |
| Total redox | NADH + NADPH + Fd^2-^ | -1.125442443 | -1.454675566 |
| ATP | (1 * v1) + (1 * v2) + (1 * v7) | 2.164259833 | 2.343495278 |
| ATP (v7 only) | (1 * v7) | 0.560019485 | 0.686697271 |
| Acetyl-CoA | (1 * v6) + (1 * v7) | 0.785349989 | 0.733130305 |
